# Predicting the necessity of anterior communicating artery division in the bifrontal basal interhemispheric approach

**DOI:** 10.1007/s00701-016-2884-3

**Published:** 2016-06-22

**Authors:** Shinichiro Teramoto, Helmut Bertalanffy

**Affiliations:** 1 Department of Neurosurgery, Juntendo University School of Medicine, 2-1-1 Hongo, Bunkyo-ku, 113-8421 Tokyo, Japan; 2 International Neuroscience Institute Hannover, Rudolf-Pichlmayrstrasse 4, D-30625 Hannover, Germany

**Keywords:** Anterior communicating artery, Basal interhemispheric approach, Brain tumor, Operative technique

## Abstract

**Background:**

The anterior communicating artery (ACoA) often limits surgical exposure in the anterior interhemispheric approach. Although division of the ACoA has been proposed occasionally, it is rarely practiced, and criteria for such a surgical maneuver remain unknown. Our purpose was to identify key factors that allow for predicting the necessity of controlled ACoA division in the bifrontal basal interhemispheric approach.

**Method:**

Twenty-two consecutive patients who underwent surgery via the bifrontal basal interhemispheric approach for removal of various pathologic brain lesions were examined. First, tumors were dichotomized into central and lateral lesions. Next, three tumor parameters were compared between cases with and without ACoA division in each, the central and lateral lesion groups, respectively: tumor volume, tumor depth (defined as distance between the ACoA and posterior tumor margin) and tumor laterality angle (defined as the geometric angle between the lateral tumor margin and sagittal midline).

**Results:**

Tumor volume was not related in a statistically significant manner to ACoA division in both the central (*P* = 0.06) and lateral (*P* = 0.13) lesion groups, respectively. However, tumor depth was significantly correlated with ACoA division in the central lesion group (*P* = 0.01), whereas in the lateral lesion group, the tumor laterality angle showed a significant correlation with ACoA division (*P* = 0.04).

**Conclusions:**

Our results suggest that controlled ACoA division may be required in central lesions with a depth of 38 mm or more and in lateral lesions with an angle of 23 degrees or more as defined in this study. Two key factors were thus identified that may predict the necessity of controlled ACoA division before surgery.

## Introduction

The basal interhemispheric approach is an ideal procedure to access tumors located in the suprasellar region, the anterior third ventricle and basal cisterns. It provides a wide surgical field without sacrificing important bridging veins. As a disadvantage, the anterior communicating artery (ACoA) often limits the surgical exposure in the anterior interhemispheric fissure. Especially in retrochiasmatic tumors, the ACoA, which is usually located anterior to the tumor, not only limits but also obstructs the surgical corridor in the basal interhemispheric approach. While some authors have proposed ACoA division if necessary [2, 11, 12], it appears that interrupting the ACoA intentionally during a surgical procedure has only rarely been practiced. It is well known that the ACoA is encountered in a great number of anatomic variations [6, 10, 11]. In some cases with normal ACoA, it may not be easy to divide the artery because of its tortuous form or because it harbors several perforating branches. Also, it must be taken into account that in the limited anterior interhemispheric area, surgical manipulation may cause damage not only to the frontal lobes but, particularly in pediatric patients with fragile arterial wall, may also induce inadvertent ACoA rupture as we have experienced in two cases. Such arterial rupture may potentially be associated with serious problems for the patient. Consequently, predicting the necessity of controlled ACoA division before surgery appears meaningful and may help reduce the surgical risk. In this context we have investigated three tumor parameters presented in this study. To our knowledge, no previous report has ever proposed similar predictors.

## Materials and methods

Twenty-two consecutive patients suffering from various brain tumors who underwent surgery via the bifrontal basal interhemispheric approach between 2009 and 2015 were examined. Pathological lesions included pilocytic astrocytoma, pilomyxoid astrocytoma, rosette-forming glioneuronal tumor, optic glioma, craniopharyngioma, central neurocytoma, cavernoma and hypothalamic hamartoma (Table 1). The senior author carried out all surgical procedures (H.B.). There were 14 females and 8 males whose ages ranged from 9 months to 42 years.

**Table 1:** Summary of clinical and morphological patient characteristics*

Lesion	ACoA division	Case	Age	Sex	Diagnosis	Location	Volume (ml)	Depth (mm)	Laterality angle (°)	ACoA anomaly	Removal	mRS
Central	Without	1	9 months	F	Pilocytic astrocytoma	Suprasellar	21.2	21.1	19.5	No	TR	1
		2	3 years	M	Pilomyxoid astrocytoma, Re	Suprasellar	16.5	24.1	10.6	No	TR	2
		3	11 years	F	Pilocytic astrocytoma, Re	Suprasellar	18.9	25.3	12.2	No	TR	1
		4	32 years	F	RGNT	Suprasellar	7.3	28.7	11.1	No	TR	1
		5	33 years	F	Craniopharyngioma	Sellar-suprasellar	4.5	20.8	10.9	Aneurysm	TR	1
		6	17 years	F	Craniopharyngioma	Sellar-suprasellar	15.6	32.0	11.8	Fenestration	TR	1
		7	6 years	M	Optic glioma, Re	Optic chiasm	3.9	12.0	10.0	No	NT	2
		8	30 years	F	Craniopharyngioma	Sellar-suprasellar	6.1	20.0	9.0	No	TR	1
	With	9	20 years	F	Cavernoma	Midbrain	10.7	42.5	11.6	No	TR	2
		10	2 years	F	Pilomyxoid astrocytoma	Suprasellar	87.8	27.5	28.0	No	NT	3
		11	5 years	F	Craniopharyngioma	Sellar-suprasellar	37.0	26.0	13.8	No	TR	1
		12	22 years	F	Cavernoma	Midbrain	5.9	37.2	8.3	Short ACoA	TR	1
		13	20 years	M	Central neurocytoma	Suprasellar	46.5	51.9	14.7	Short ACoA	TR	1
		14	16 years	M	Pilocytic astrocytoma, Re	Suprasellar	68.1	44.4	21.9	No	NT	2
Lateral	Without	15	7 years	M	Hypothalamic hamartoma, Re	Hypothalamus, left	4.2	17.3	15.0	No	NT	1
		16	35 years	F	Cavernoma	Midbrain, right	2.6	34.4	7.1	Fenestration	TR	1
		17	42 years	F	Cavernoma	Midbrain, left	2.5	32.4	11.8	No	TR	1
		18	3 years	F	Cavernoma	Hypothalamus, left	2.1	15.9	11.4	No	TR	1
		19	15 years	M	Cavernoma	Midbrain, left	5.5	34.1	9.7	Short ACoA	TR	1
		20	33 years	M	Cavernoma	Thalamus, right	3.3	19.2	11.8	No	TR	1
	With	21	17 years	F	Pilocytic astrocytoma	Thalamus, right	31.8	54.8	21.3	Fenestration	ST	2
		22	4 years	M	Pilocytic astrocytoma	Thalamus, left	22.1	28.4	25.1	No	NT	2

*ACoA = anterior communicating artery; mRS = modified Rankin Scale; TR = total removal; NT = near total removal; ST = subtotal removal; Re = recurrence; RGNT = rosette-forming glioneuronal tumor

**Fig. 1:**
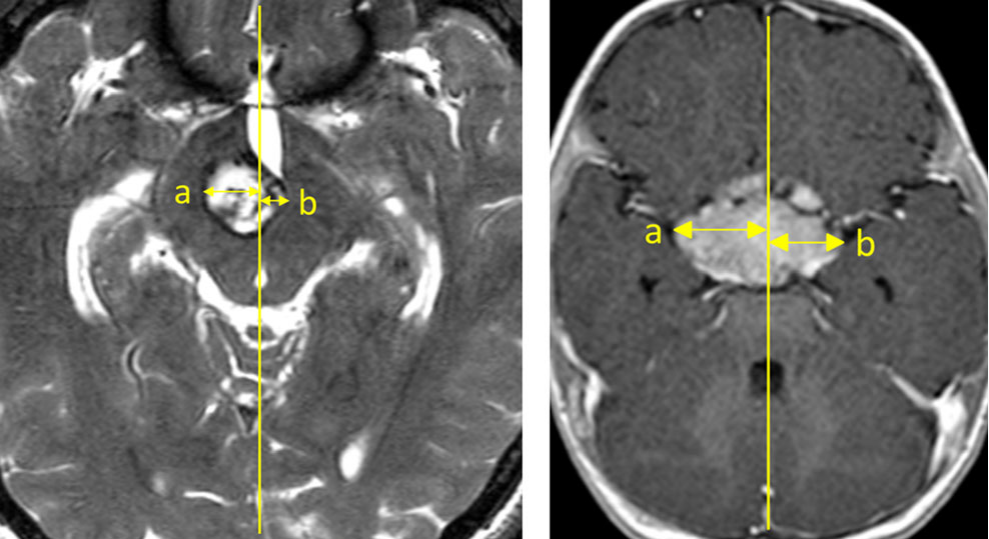
Definition of *central* and *lateral* lesion. A lesion was defined as “lateral” (*left*) when the ratio between its larger (**a**) and smaller horizontal width (**b**) measured on each side of the sagittal midline equaled or exceeded 2:1 (in this case, a = 12.7 mm; b = 5.1 mm; a/b ratio = 2.5). A lesion was determined as “central” (*right*) when this ratio was below 2:1 (in this case, a = 21.5 mm; b = 16.9 mm; a/b ratio = 1.3)

In a first step tumors were dichotomized into a central and lateral lesion group, respectively. A lesion was defined as “lateral” when the ratio between its larger and smaller horizontal width measured on each side from the sagittal midline equaled or exceeded 2:1; pathologies with a ratio below 2:1 were determined as “central” lesions (Fig. 1).

**Fig. 2:**
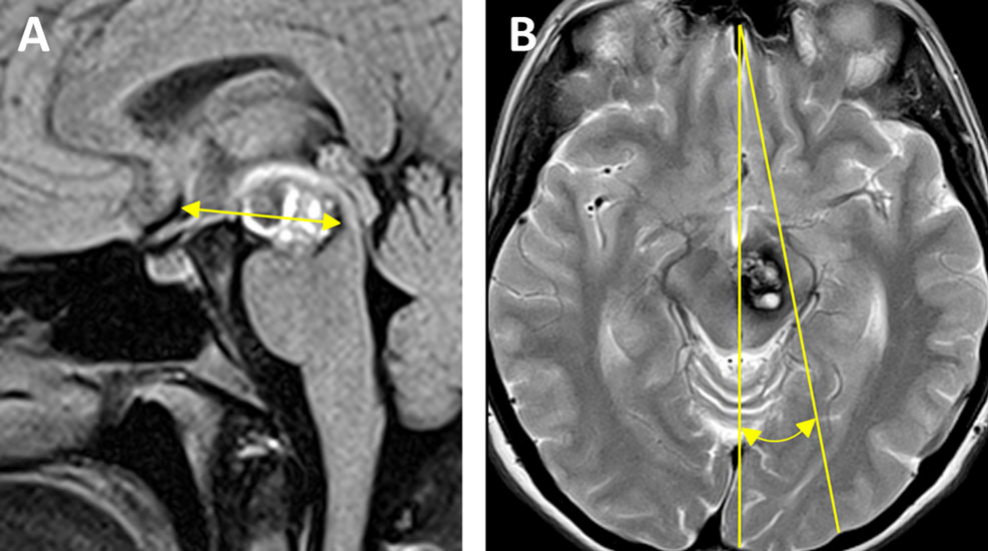
Definition of the tumor *depth* and *laterality angle*. **a** Tumor depth was defined as the distance between the anterior communicating artery and posterior tumor margin on the sagittal MRI slice with the largest tumor extent. **b** Tumor laterality angle was determined as the geometric angle between the sagittal midline and a line connecting the anterior margin of the frontal lobe in the midline with the most lateral tumor margin on the axial MRI slice with the largest tumor extent

In a next step, three tumor properties (volume, depth and laterality angle) were compared between patient groups with and without ACoA division in each, the central and lateral lesion group, respectively (Table 2). Tumor volume was calculated using the ABC/2 method on magnetic resonance imaging (MRI) [4, 14]. Tumor depth was evaluated by defining this parameter as the distance between the ACoA and posterior tumor margin on the sagittal MRI slice containing the largest antero-posterior lesion extent (Fig. 2a). All distance measurements were directly performed on a viewing monitor using the neuroradiological software. To assess tumor laterality, a third parameter named the tumor laterality angle was analyzed. This parameter was defined as the geometric angle between the sagittal midline and a line that connects the most anterior margin of the frontal lobe in the midline with the most lateral tumor margin measured on the axial MRI slice with the largest tumor extent (Fig. 2b).

**Table 2:** Comparison between patient groups with and without ACoA division according to tumor volume, depth and laterality angle*

Lesion		Division of ACoA		*P* Value
		Without	With	
Central	Volume (ml) ± SD	11.7 ± 6.5	42.7 ± 29.2	0.06
	Depth (mm) ± SD	23.0 ± 5.7	38.3 ± 9.2	0.01†
	Laterality angle (°) ± SD	11.9 ± 3.0	16.4 ± 6.6	0.20
Lateral	Volume (ml) ± SD	3.4 ± 1.2	26.9 ± 4.8	0.13
	Depth (mm) ± SD	25.6 ± 8.2	41.6 ± 13.2	0.43
	Laterality angle (°) ± SD	11.1 ± 2.4	23.2 ± 1.9	0.04†

*ACoA = anterior communicating artery; SD = standard deviation

†Statistically significant at *P* < 0.05

All statistical analyses were conducted utilizing the EZR (Saitama Medical Centre, Jichi Medical University, Saitama, Japan), which is a graphical user interface for R (The R Foundation for Statistical Computing, Vienna, Austria, version 2.13.0) [3]. Average values for groups were given as mean and standard deviation or as median and interquartile range (IQR). The data were analyzed by the unpaired t-test and Mann-Whitney U-test. Statistical significance was defined as *P* < 0.05.

### Surgical technique with ACoA division in tumors of the anterior third ventricle

The patient was positioned supine with the neck in slight extension. A bicoronal skin incision was made behind the hairline, and subperiosteal dissection was extended toward the supraorbital ridge while protecting the supraorbital nerves. A bifrontal craniotomy involving resection of the nasal part of the frontal bone was performed. If the frontal sinus was exposed, the sinus mucosa was detached and cauterized. The frontal dura was opened transversely from lateral to medial, and the ligated superior sagittal sinus and proximal falx were divided.

After dural opening, both olfactory nerves were preserved by dissecting them from the base of the frontal lobe. The anterior interhemispheric fissure was separated toward the optic chiasm, the ACoA and the entire lamina terminalis up to the genu of the corpus callosum. Relaxation of frontal lobes by opening the chiasmatic cistern and lamina terminalis enlarged the surgical field anterior to the third ventricle.

The tumor portion within the anterior third ventricle was removed from the optic chiasm superiorly, inferiorly or laterally. In cases of excessive superior tumor extension, resection of the anterior commissure occasionally became necessary. If the tumor was located very deeply or extended far laterally, division of the ACoA was considered to enlarge the surgical corridor. This widened the surgical field and minimized the risk for inadvertent vascular injuries. The ACoA was divided following its occlusion with two vascular mini clips while preserving its perforating branches. Blood flow of the A1 and A2 segments of the anterior cerebral arteries (ACAs) was verified with a microvascular Doppler device before and after ACoA division. Although dividing the ACoA was more difficult in cases with ACoA anomalies such as short segment or fenestration, it was possible to divide the ACoA following clip occlusion in all patients of our series. According to our experience preoperative angiography was not required for ACoA division because we considered it impossible to precisely identify perforating ACoA branches or an anomalous ACoA on general angiograms. Assessment of a hypoplastic A1 segment or other variations was easily evaluated by intraoperative inspection.

The tumor was gently and, whenever possible, totally removed with care for important surrounding structures such as the optic chiasm and tracts, pituitary stalk and hypothalamus.

The dura was closed in a watertight fashion. The exposed frontal sinus was covered using a vascularized pericranial flap followed by bone replacement and skin closure.

### Illustrative cases

#### Case 9 (Table 1)

**Fig. 3:**
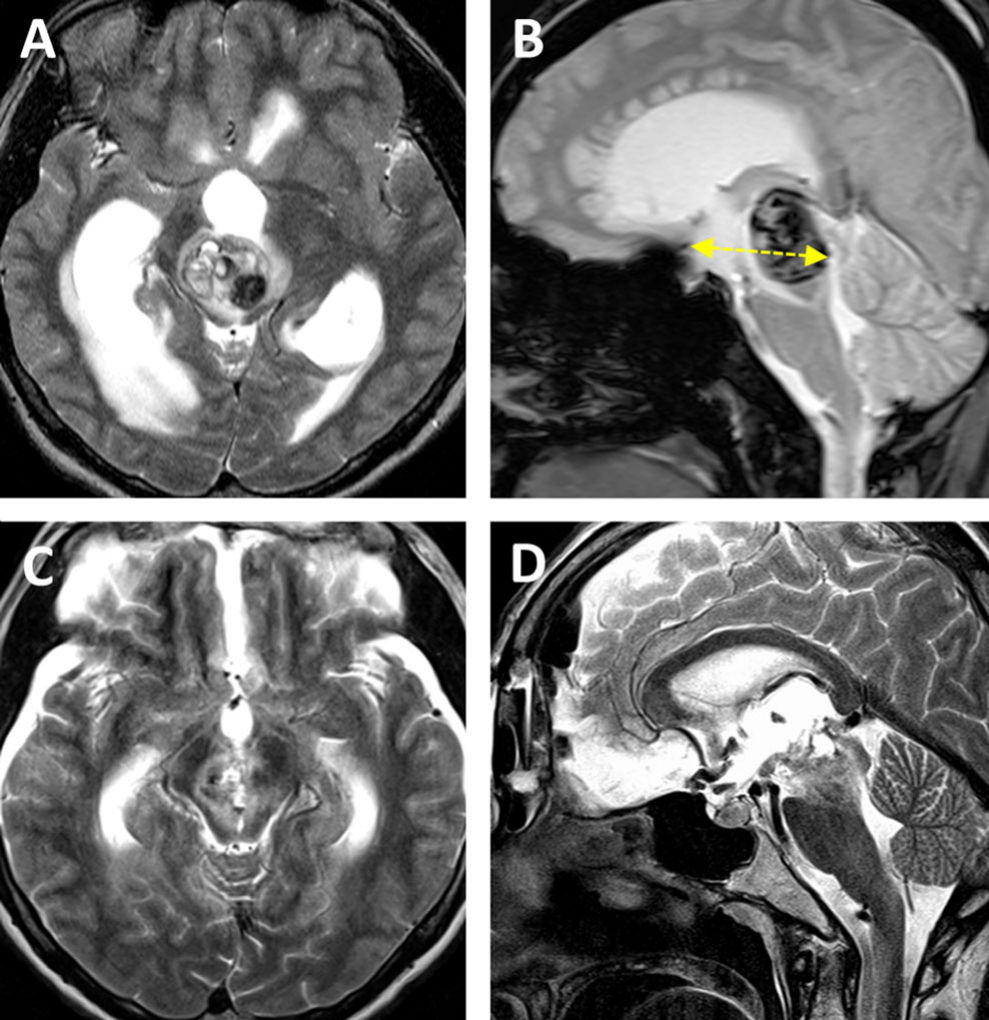
Pre- and postoperative MRI of case 9. **a**, **b** Preoperative MRI of a hemorrhagic midbrain cavernoma that was classified as “central lesion.” The yellow dotted line shows the tumor depth (42.5 mm). **c**, **d** Postoperative MRI after total removal of the vascular lesion

This 20-year-old female without a prior medical history presented with suddenly disturbed and gradually worsening consciousness and left hemiparesis. MRI revealed a fresh intraaxial hemorrhage in the midbrain typical for cavernoma bleeding with consecutive acute obstructive hydrocephalus. The lesion was situated in the ventral mesencephalic midline and was noted to be 10.7 mm^3^ in volume, of 42.5 mm depth and of 11.6 ° tumor laterality angle (Fig. 3a, b). Removal of the hemorrhagic lesion via the bifrontal basal interhemispheric approach was accomplished successfully 3 days after placement of a right external ventricular drain.

**Fig. 4:**
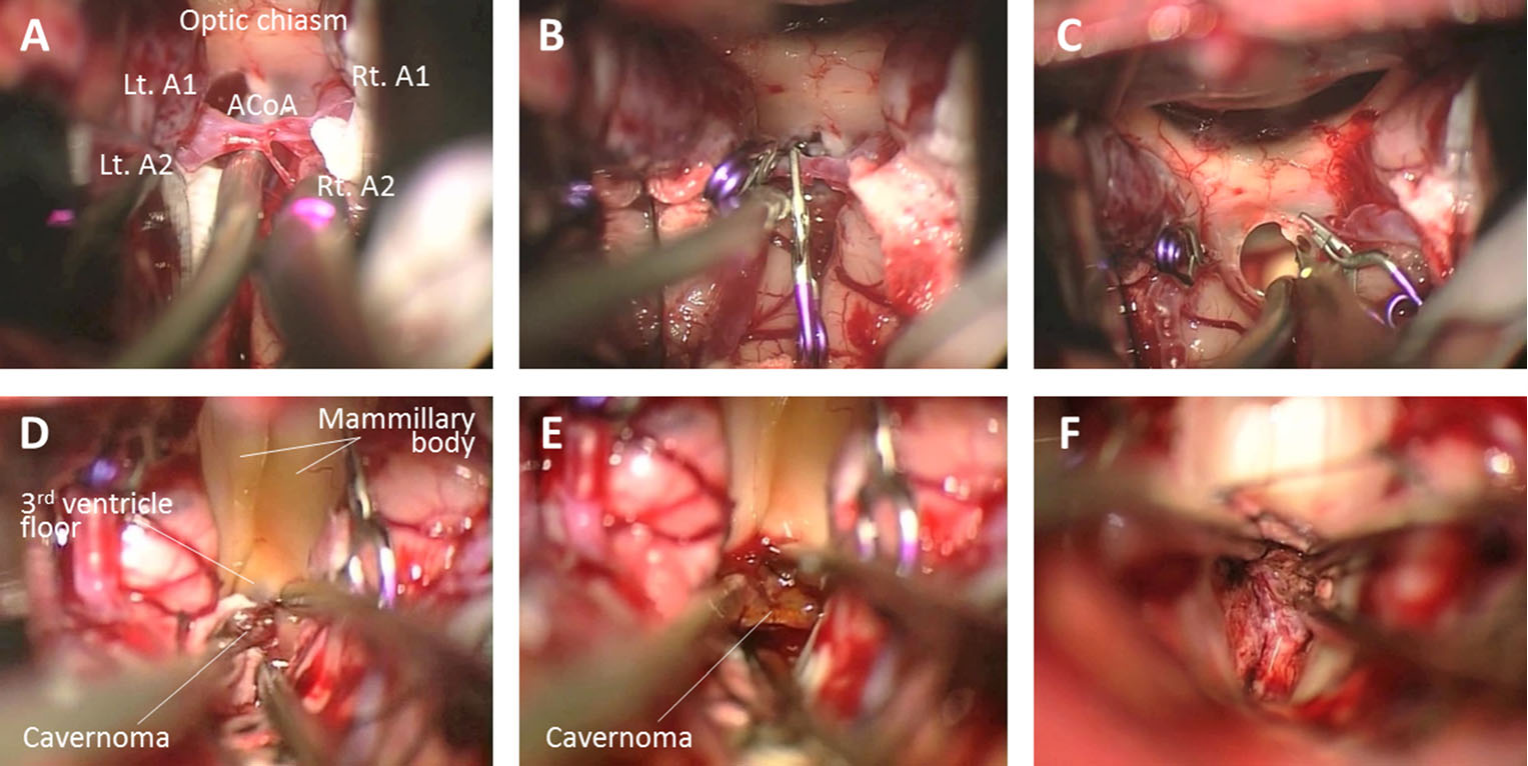
Intraoperative photographs of dividing the anterior communicating artery (ACoA) of case 9. **a–c** The ACoA was occluded using mini clips while preserving the perforating branch of the ACoA and was subsequently divided between the clips. **d–f** After cutting the lamina terminalis longitudinally, the floor of the third ventricle was opened between the mammillary bodies. The exposed midbrain cavernoma was entirely removed by piecemeal resection. Rt, right; Lt, left; A1, A1 segment of the anterior cerebral artery; A2, A2 segment of the anterior cerebral artery

Following skin incision and craniotomy, the anterior interhemispheric fissure was separated to the anterior wall of the third ventricle. For sufficient exposure of the anterior brainstem through the third ventricle, ACoA division was estimated beneficial and necessary during surgery. The ACoA was occluded with mini clips and subsequently divided while preserving a perforating branch (Fig. 4a–c). Next, the lamina terminalis was cut longitudinally, and the floor of the third ventricle was opened between the mammillary bodies leading directly to this midbrain vascular malformation. The cavernoma was dissected free and removed in piecemeal fashion. Tiny arterial feeders were repeatedly coagulated and cut at the interface between the lesion and parenchymal tissue, and the hematoma and cavernoma were entirely removed (Fig. 4d–f).

The postoperative course was uneventful. The patient’s consciousness and hemiparesis rapidly improved. Hydrocephalus regressed, and shunt placement was not necessary. The patient was discharged on foot, and there were no neurological or cognitive deficits at follow-up 3 months after surgery. Postoperative MRI demonstrated total removal of the intraaxial hematoma and vascular lesion (Fig. 3c, d).

#### Case 14 (Table 1)

**Fig. 5:**
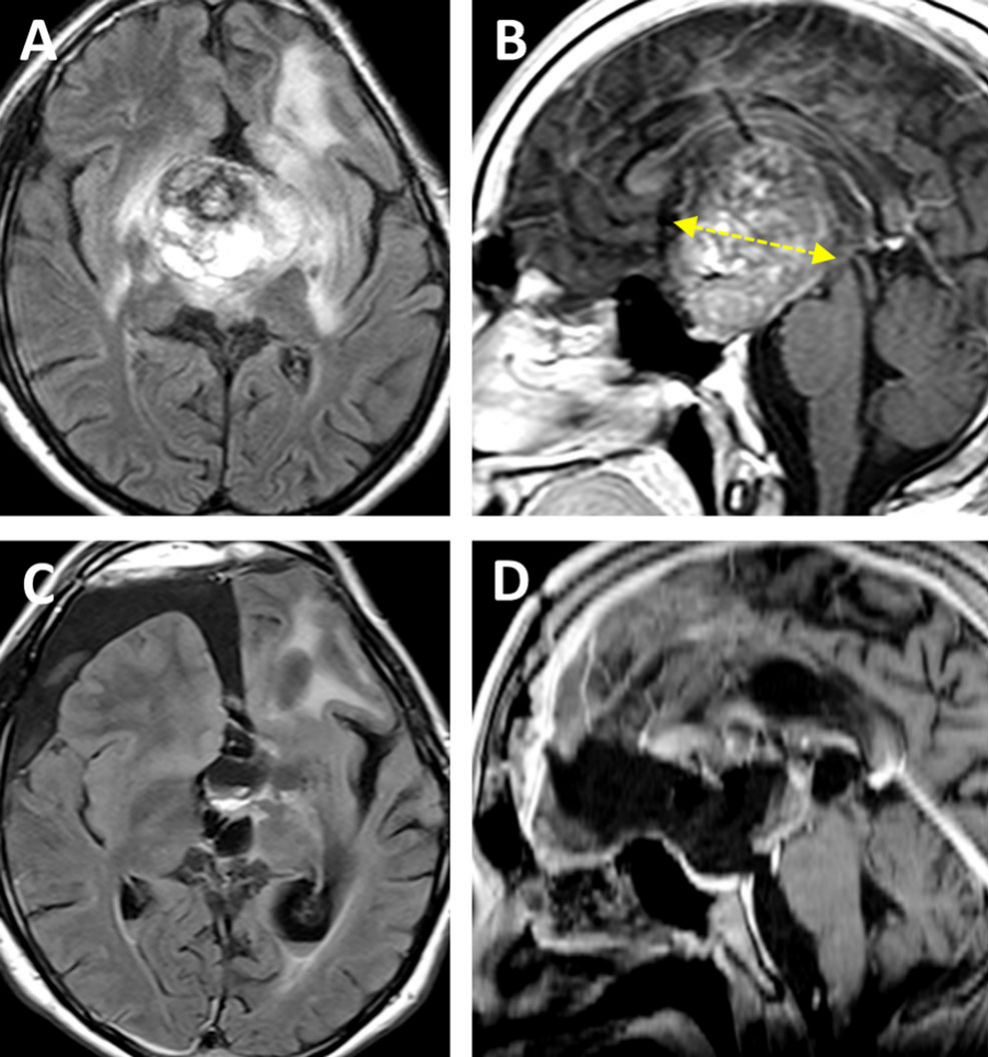
Pre- and postoperative MRI of case 14. **a**, **b** Preoperative MRI of a recurrent pilocytic astrocytoma that was classified as “central lesion.” The yellow dotted line shows the tumor depth (44.4 mm). **c**, **d** Postoperative MRI after near total tumor removal

This 16-year-old male who previously underwent surgical resection of a suprasellar tumor at the age of 4 years via a left-sided pterional approach was referred to our hospital. In spite of two cycles of adjuvant chemotherapy for treatment of the diagnosed hypothalamic pilocytic astrocytoma, the tumor massively recurred over the following years. At admission, the patient was alert but suffered from panhypopituitarism and severe left-sided visual impairment equaling almost blindness in this eye. Preoperative MRI demonstrated a giant tumor occupying the suprasellar space that extended into the left parahippocampal gyrus and lateral ventricles (Fig. 5a, b). Tumor volume amounted to 68.1 mm^3^, its depth to 44.4 mm and the laterality angle to 21.9 °. Surgical removal was considered the only reasonable treatment modality at this stage.

**Fig. 6:**
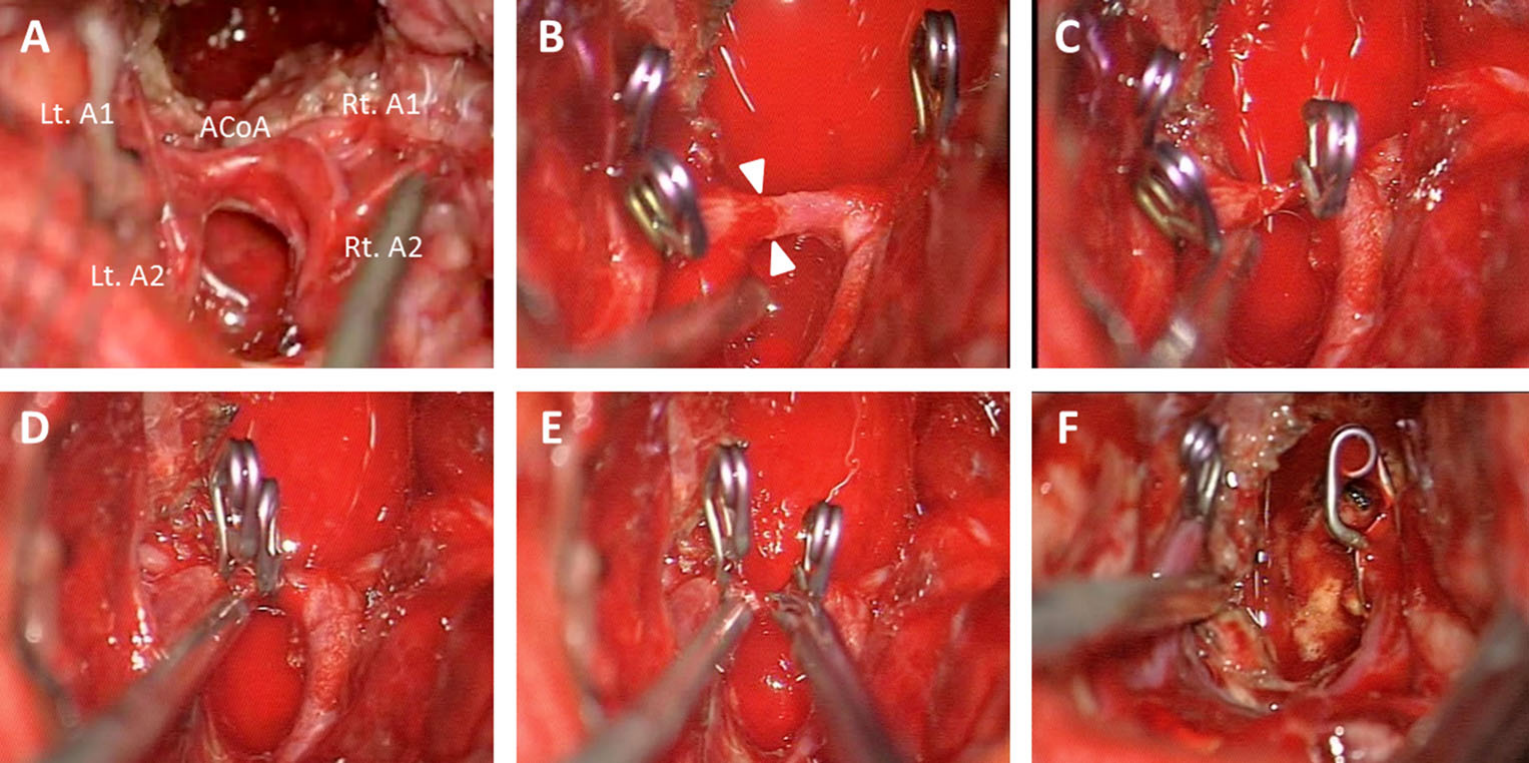
Intraoperative photographs of dividing the anterior communicating artery (ACoA) of case 14. **a–f** Because the ACoA was unexpectedly lacerated, both A1 segments of the anterior cerebral artery (A1) were temporarily occluded for flow control. The lacerated point (*between the white arrowheads*) of the ACoA was trapped with mini clips and subsequently divided. Rt, right; Lt, left; A2, A2 segment of the anterior cerebral artery

A bicoronal skin incision, bifrontal craniotomy and basal interhemispheric approach were undertaken in a routine fashion. First, the tumor involving the optic chiasm was removed, and the pituitary stalk was identified below the chiasm. The ACA, ACoA and lamina terminalis were gradually exposed. Opening the lamina terminalis permitted wide access to the anterior third ventricle and allowed for significantly debulking the retrochiasmatic tumor portion that had filled this area up to the basilar bifurcation and P1 segment of the posterior cerebral artery. While the tumor was meticulously resected despite its firm adherence to the hypothalamus and posterior circulation vessels, the ACoA was unexpectedly lacerated by tensile stress. Since repair of the lacerated ACoA was impossible, it was decided to divide the ACoA and thus maintain the blood supply in both A2 segments of the ACA. Following temporary occlusion of both A1 segments, the lacerated point of the ACoA was trapped with mini clips and subsequently divided (Fig. 6a–f). Vascular patency was repeatedly controlled with a micro Doppler device. Removal of the residual tumor could then be readily accomplished. Near total tumor resection and normal perfusion in both A2 vascular territories were confirmed and documented on intraoperative MRI (Fig. 5c, d). The optic chiasm and tracts, pituitary stalk and hypothalamus remained intact.

The postoperative course was uneventful, and the patient continued the medical treatment of panhypopituitarism. At follow-up no additional neurological or cognitive deficits were noted.

## Results

Clinical and morphological characteristics of all patients are summarized in Table 1. Fourteen patients harbored a central and eight a lateral lesion. Of all 22 patients, 8 (6 with central and 2 with lateral lesions) underwent ACoA division, while 14 individuals (8 with central and 6 with lateral lesions) did not require such arterial interruption. In one patient, a 3-mm ACoA aneurysm was found incidentally and treated by surgical clipping after tumor removal (case 5).

Total removal was achieved in 16 patients, near total removal (more than 90 % of tumor volume) in 5 and subtotal removal (between 50 and 90 % of tumor volume) in 1 individual. Fortunately, no serious early or late complications related to the surgical procedure or ACoA division occurred. Median modified Rankin Scale (mRS) score at discharge was 2 (IQR, 1-2) in patients who underwent ACoA division and was 1 (IQR, 1-1) in those without arterial interruption. No significant difference in mRS score between ACoA division and non-division patient groups was found at discharge (*P* = 1.22).

Conversely, comparison of the three tumor properties between the ACoA division and non-division groups in each central and lateral lesion, respectively, resulted in interesting findings. Tumor volume was not statistically related to ACoA division in either the central (*P* = 0.06) or lateral (*P* = 0.13) lesion groups (Table 2). However, in the central lesion group, tumor depth was significantly correlated with ACoA division (*P* = 0.01) (Table 2), while in the lateral lesion group, the tumor laterality angle showed a significant correlation with ACoA division (*P* = 0.04) (Table 2).

## Discussion

Intentionally dividing the ACoA during a surgical procedure is commonly avoided to preserve perforating branches emerging from the ACoA, which can act as the main feeders of the infundibulum, optic chiasm and anterior hypothalamus [1, 9]. Injury of such perforating ACoA branches may cause endocrine dysfunction, cognitive impairment and psychiatric disorder [5, 7, 8, 13]. Additionally, dividing the ACoA, of which approximately 60 % show anatomic variations [6, 10, 11], may not be easy, particularly in children with a very short ACoA segment. Nevertheless, a few authors have mentioned the main advantage of controlled ACoA division, namely obtaining a wider surgical exposure in the anterior interhemispheric fissure [2, 11, 12]. This arterial interruption was not associated with complications in their reports. Similarly, in our patient series a favorable outcome and lack of complications were equally noted in patients who did or did not undergo ACoA division. Paying sufficient attention to the usually singular subcallosal artery, which is the most important and largest vessel among the perforating ACoA branches [7, 10], the ACoA can safely be divided in most instances. Moreover, several perforating branches of the ACoA and their anastomoses supply the hypothalamic area abundantly [1, 10]. Such anatomical circumstances may prevent the above-mentioned complications. However, specific predictors of whether or not the ACoA should be divided during a surgical procedure have so far not been described. Previous reports dealing with this problem were all related to craniopharyngioma surgery and did not analyze the requirements of ACoA division [2, 11, 12]. In the present study we analyzed patients suffering from various histopathological types of suprasellar lesions who underwent surgery via the bifrontal basal interhemispheric approach, trying to identify specific criteria that may predict the necessity of ACoA division. In the narrow surgical corridor of the basal interhemispheric approach, tumor volume and antero-posterior and lateral extent were regarded as essential parameters that may influence the possibility of total tumor removal. While a smaller tumor size obviously facilitates removal, a large tumor extension renders surgical resection in this area more difficult. Particularly tumor portions located far behind the ACoA are not readily accessible as the ACoA often obstructs or complicates microsurgical exposure. In this context we concentrated on three morphological tumor parameters, namely tumor volume, depth and maximal lateral extent.

Contrary to our expectation, tumor volume did not arise as an adequate parameter to predict the necessity of ACoA division in either the so-called central or lateral lesion groups, respectively. Although statistical variability of the mean tumor volume due to the different lesion types might have affected our analysis, Shibuya et al. also indirectly mentioned in their publication dealing with craniopharyngioma surgery that there was no difference in tumor size between patient groups with and without ACoA division [11]. This report and our results suggest that the necessity of ACoA division may not depend on the tumor volume.

On the other hand, our results showed that intentionally dividing the ACoA before tumor removal may be required in a so-called central lesion (according to the definition in the present study) with a tumor depth close to 38 mm or a maximal lateral extension over 23 degrees of laterality angle as we have defined this parameter in the present study.

We first thought about measuring the true lateral width of the lesion to assess its lateral extent. However, the same lateral extension of a lesion located anterior to or far behind the ACoA may not have the same meaning regarding the necessity of lateral brain retraction. For this reason we preferred to assess the lesion’s laterality by measuring the “laterality angle” as we defined this parameter in the Materials and Methods section.

In summary, in the basal interhemispheric approach, the ACoA can be regarded as a significant limitation or obstruction when the posterior tumor portions extend far away from the ACoA, rendering tumor depth an essential parameter. Likewise, exposure of lesions with significant lateral extent within the anterior interhemispheric fissure may lead to excessive tensile stress upon the ACoA and increase the risk for inadvertent vascular rupture. Tumor laterality proved, therefore, to be another important morphologic parameter that may be assessed on preoperative imaging to help predict the necessity of controlled ACoA division before tumor removal.

## Conclusions

Intentional and controlled ACoA division can be a beneficial technique in the basal interhemispheric approach and allow for safe and total tumor removal. The present study identified two key factors that can be easily assessed on preoperative MR imaging (tumor depth and maximal lateral extent) and that may help predict the necessity of ACoA division before surgery. However, the final decision whether or not to divide the ACoA should always be taken during the surgical procedure after careful intraoperative inspection without the need for preoperative angiography.
